# Retraction: An African Ancestry-Specific Allele of *CTLA4* Confers Protection against Rheumatoid Arthritis in African Americans

**DOI:** 10.1371/annotation/80bd7285-9d2d-403a-8e6f-9c375bf977ca

**Published:** 2009-12-01

**Authors:** James M. Kelley, Laura B. Hughes, Jeffrey D. Faggard, Maria I. Danila, Monica H. Crawford, Yuanqing Edberg, Miguel A. Padilla, Hemant K. Tiwari, Andrew O. Westfall, Graciela S. Alarcón, Doyt L. Conn, Beth L. Jonas, Leigh F. Callahan, Edwin A. Smith, Richard D. Brasington, David B. Allison, Robert P. Kimberly, Larry W. Moreland, Jeffrey C. Edberg, S. Louis Bridges

In June 2009, one of our colleagues, Dr. Robert Plenge, pointed out to us that he and his colleagues reviewed this article and were concerned that our finding may reflect an artifact in the minor allele (G) frequency of the rs231778 SNP in our control group. Dr. Plenge graciously agreed to genotype this SNP and other SNPs in the CTLA4 gene in our African-American patients and controls using a Sequenom iPlex Pool assay. In addition, we repeated the genotyping of the rs231778 SNP in a subset of the African-American controls reported in our study using the same platform as previously reported (Applied Biosystems TaqMan Allelic Discrimination Assays on an ABI 7900HT Genetic Analyzer) with new reagents. In these repeat experiments, we found no difference in the minor allele frequency of the rs231778 SNP between African-American patients (0.060) and controls (0.065) (p=0.607), validating the concern that our finding represented an artifact in genotyping.

The major conclusion of our paper, that an African ancestry-specific allele of the CTLA4 gene confers protection against RA in African Americans, is therefore invalid, and the paper must be retracted. The decision to retract this paper was reached in consultation with the journal's editors and the purpose of the retraction is to correct the literature.

We wish to acknowledge Dr. Plenge's collegiality and his generosity as he performed the Sequenom genotyping at his expense. Based on the collaboration that grew out of his identification of our error, we are currently preparing a new article describing the role of the CTLA4 gene and other candidate genes in African-Americans with RA.

